# Five-year trends in antibiotic use and microbiology of hospitalized diabetic foot infections in the Bronx

**DOI:** 10.1017/ash.2026.10824

**Published:** 2026-09-09

**Authors:** Jovan Milosavljevic, Ava Tsapatsaris, Erika Orner, Alexander Miller, Mei Chang, Shiming Xu, Alyson K. Myers, Johanna P. Daily

**Affiliations:** 1 Albert Einstein College of Medicine, USA; 2 Harvard Medical School, USA; 3 University of Massachusetts Chan Medical School, USA

## Abstract

**Background:**

Diabetic foot infections (DFIs) are a major cause of hospitalization and antibiotic use, yet variation in diagnostic sampling and empiric therapy persists. We evaluated microbiologic findings and antibiotic utilization in hospitalized patients with DFIs over a five-year period.

**Methods:**

We conducted a retrospective cohort study of adults hospitalized with diabetic foot ulcers who received systemic antibiotics at Montefiore Medical Center (Bronx, NY) from 2016–2021. We assessed timing of wound culture collection, microbiologic results, and antibiotic use. Associations between time to culture and length of stay (LOS), and temporal trends in prescribing, were analyzed using linear regression.

**Results:**

Among 637 patients (mean age 59 ± 13 years; 70% male; 53% Hispanic), median LOS was 7 days (IQR 4–11). Wound cultures were obtained in 450 patients (71%), typically on hospital day 1 (IQR 0–2). Time to first culture was positively associated with LOS (Pearson *r* = .47, *P* < .001) with each additional day associated with 1.18 day increase in LOS (*P* < .001). Methicillin susceptible *Staphylococcus aureus* (MSSA) and *Streptococcus* species predominated in wound cultures, while methicillin resistant *S. aureus* (MRSA) and *Pseudomonas aeruginosa* were each identified in <10% of cases. Among patients with blood cultures, 26% had bacteremia, most commonly MSSA and MRSA bacteremia occurred in 6.5%. Despite this, empiric anti-MRSA and antipseudomonal therapy were initiated in 91% and 80% of patients, respectively. Over the study period, vancomycin use declined (*P* < .01), and the use of non-antipseudomonal cephalosporins increased (*P* = .04).

**Conclusions:**

In this large cohort of hospitalized patients with DFIs, MSSA predominated and resistant organisms were infrequent; however, empiric broad-spectrum antibiotic use was common. Earlier culture acquisition and alignment of empiric therapy with local microbiologic epidemiology represent key opportunities for antimicrobial stewardship.

## Introduction

Diabetic foot ulcers (DFU) are a major complication of diabetes and the leading cause of limb loss in people with diabetes.^1^ The lifetime risk of DFU is estimated to be between 15% and 25% among individuals with diabetes.^2^ DFUs are associated with increased emergency department utilizations, hospitalizations and mortality.^3^ Between 2005 and 2010, more than one million people with diabetic foot complications presented to emergency departments in the United States, with over 80% requiring inpatient admission.^4^

DFUs arise from the combined effects of peripheral neuropathy and peripheral vascular disease, which increase susceptibility to pressure-related injury and impaired wound healing. Once present, ulcers are highly prone to infection, with approximately 50%–60% progressing to diabetic foot infection (DFI), often resulting in tissue destruction, bacteremia, and amputation.^5^ Optimal management of DFI includes prompt and appropriate specimen collection, surgical debridement when indicated and targeted antimicrobial therapy.^1^

Despite the availability of guideline recommendations, substantial variation persists in diagnostic sampling and empiric antibiotic selection in clinical practice, particularly in the inpatient setting. The objective of this study was to characterize the timing and type of specimen collection, microbiologic findings, and antibiotic use among adults hospitalized with DFI from 2016 to 2021. By defining these patterns, we aim to identify opportunities for antimicrobial stewardship and inform more standardized approaches to DFI management.

## Methods

### Study design and population

We conducted a retrospective cohort study of hospitalized adults to evaluate the timing of wound sampling, characterize the microbiologic profile of DFIs, assess antibiotic utilization patterns and examine temporal trends in antibiotic use. This study was a sub-study of patients with DFU admitted to Montefiore Medical Center (MMC) in Bronx, New York between June 1, 2016, and May 31, 2021.6 The Bronx has a population of over 1.3 million residents, most of whom identify as Hispanic (55%) or Non-Hispanic Black (29%), with a poverty rate of 26.9%.^7,8^

Patients were classified as having a DFI if they received systematic antibiotics during hospitalization, an operational definition that may overestimate true infection. Patients with concurrent infectious conditions at the index hospitalization were not excluded. Antibiotic use reflected all inpatient systemic antibiotic therapy. For patients with multiple admissions, only the index admission was included to ensure consistent comparison of microbiologic findings and antibiotic use. The study was approved by the Institutional Review Board of Albert Einstein College of Medicine (IRB number 2022-14264).

### Study end points

Study end points were descriptive measures of microbiology and antimicrobial use among patients hospitalized with DFI: (1) timing of wound culture collection; (2) microbiological profiles based on wound and blood results; (3) antibiotic exposure, measured as days of therapy (DOT); and (4) temporal trends in antibiotic prescribing.

### Data collection

Data were extracted from Epic® electronic health record (EHR). Patients with DFUs were identified using ICD-10 codes E10.621 (type 1 diabetes mellitus with foot ulcer), and E11.622 (type 2 diabetes mellitus with foot ulcer) as previously described.6 Extracted variables included demographics characteristics, admission and discharge dates, antibiotic orders, and microbiologic data, including specimen source and collection date.

#### Microbiology

All patients were admitted through the emergency department, and clinical data were collected from the initial admission evaluation and throughout the hospitalization. The interval from hospital admission to the first wound specimen collection was recorded, with the day of admission designated as day 0. ￼￼Microbiological identification was performed by the clinical microbiology laboratory at MMC using standard culture-based methods and routine laboratory identification procedures.^9^ Specimens were collected by wound swab, abscess aspirate, or tissue biopsy..

Organisms isolated from wound swabs that are considered pathogenic included *Staphylococcus aureus* and other bacteria typically associated with DFI. Organisms typically regarded as skin colonizers, (ie, *Micrococcus, Bacillus, Corynebacterium,* viridans group *Streptococcus* and coagulase-negative *Staphylococcus*), were considered pathogenic only if they met predefined standardly used microbiologic criteria: (i) polymorphonuclear cells were present on Gram stain; (ii) Gram stain bacteria staining and morphology consistent with the cultured organism; and (iii) the organism demonstrated moderate or predominant growth on culture. All organisms isolated from blood cultures and fluid aspirates were considered pathogenic. Organisms recovered from tissue specimens were also considered clinically significant; however, the method and setting of tissue collection were not consistently documented in the EHR.

For patients with multiple wound cultures yielding the same organisms, each species was counted once per patient. To focus on commonly encountered pathogens, organisms identified in ≤5 patients were excluded from the primary analysis and reported separately. For blood cultures, only the first positive result per organism per patient was included.

#### Antibiotic use

Inpatient antibiotic administration orders were extracted from the EHR and categorized by antibiotic class including: β-lactam/β-lactamase inhibitor combinations (ampicillin-sulbactam and amoxicillin-clavulanic acid); anti-pseudomonal agents (cefepime, piperacillin-tazobactam, meropenem, aztreonam, gentamicin); non-antipseudomonal cephalosporins, clindamycin; daptomycin; doxycycline; fluoroquinolones; linezolid; macrolides; metronidazole; penicillins; trimethoprim/sulfamethoxazole (TMP-SMX); and vancomycin.^10^ Antibiotics were additionally classified according to the World Health Organization AWaRe framework (Access, Watch, and Reserve).^11^ Both intravenous and oral formulation antibiotics were included in the analyses.

Antibiotic use was quantified as DOT, defined as the number of calendar days on which a patient received a given antibiotic, irrespective of dose or frequency. DOTs were aggregated by antibiotic class and by AWaRe category and normalized per 1,000 patient-days. When multiple antibiotics were administered on the same day, each was counted separately to provide a total DOT.^12^ Length of therapy (LOT)was defined as the number of calendar days on which a patient received at least one systemic antibiotic, regardless of number of agents administered, and was normalized per 1,000 patient-days for analysis.^12^

### Statistical analysis

Descriptive statistics were used to summarize patient characteristics, sampling methods, microbial data and antibiotic use (Graph Prism, version 10). The association between time to wound culture collection and LOS was assessed using Pearson correlation and linear regression. Because wound culture collection dates were only available after August 2018, analyses involving time to sampling were restricted from that date onward.

Temporal trends in antibiotic use were evaluated using linear regression, with calendar year of hospitalization as the independent variable and DOT per 1,000 patient-days as the dependent variable. To assess changes in prescribing over the course of hospitalization, linear regression models were constructed with hospital day (days since admission) as the independent variable and DOT per 1,000 patient-days as the dependent variable. Separate models were fit for each antibiotic class.

All statistical tests were two-sided with a significance threshold of *P* < .05. Analyses were performed using R version 4.2.2 (R Project for Statistical Computing).

## Results

### Study population and baseline characteristics

**Figure 1.**
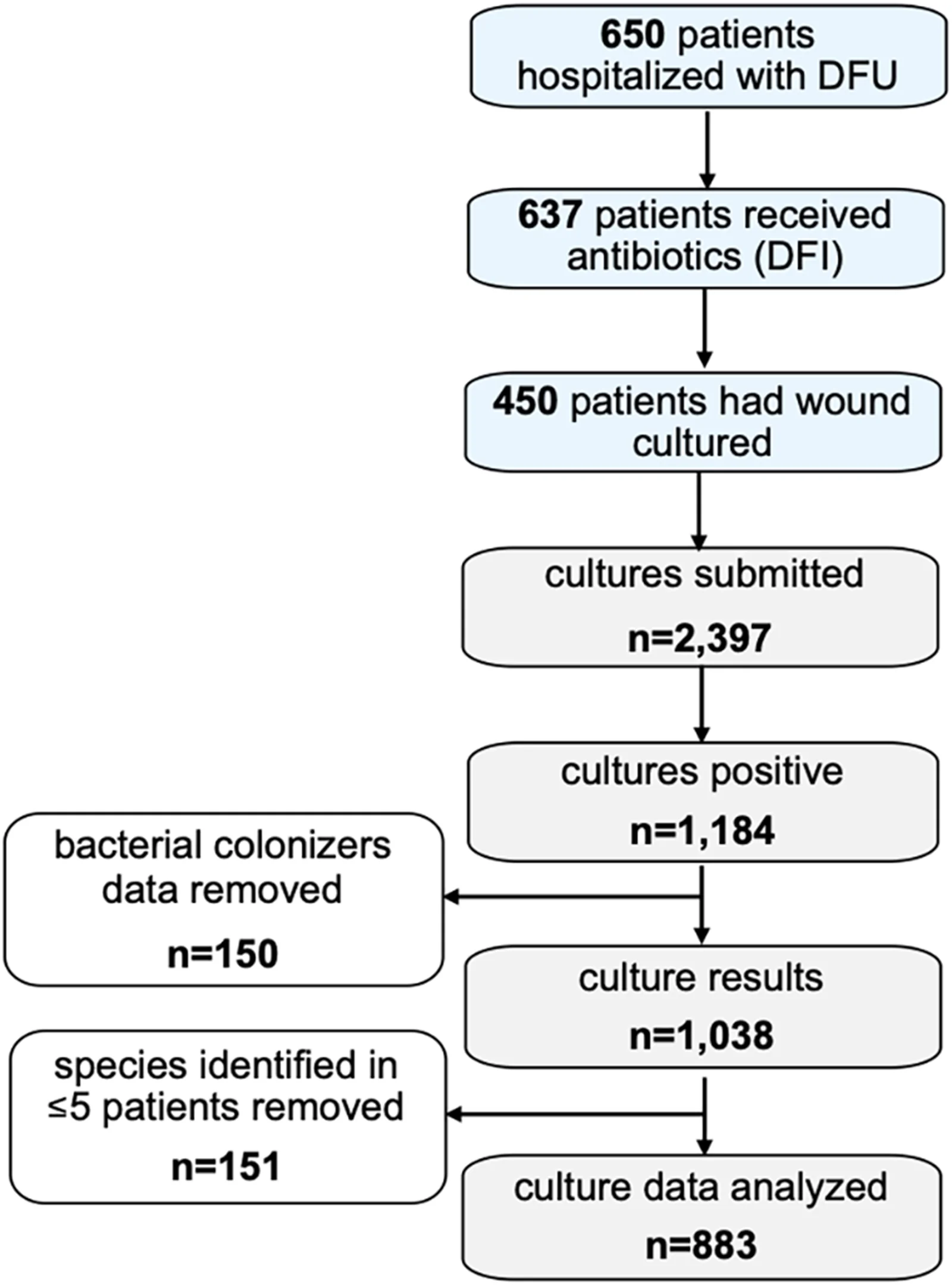
Flow diagram of patient cohort and inclusion of wound culture data for analysis. Diagram illustrating inclusion criteria for patients and wound culture data in hospitalized patients with diabetic foot infection (DFI).

DFU were diagnosed in 650 patients from the parent cohort, of whom 637 (98%) met criteria for DFI (Figure 1). The patients were predominantly male (448 [70%]) with a mean age of 59 ± 13 years, 326 (53%) identified as Hispanic and 183 (30%) Black (Table 1). The median LOS for the index DFI hospitalization was 7 days (IQR, 4–11).

**Table 1.** Demographic and clinical characteristics of patients hospitalized with diabetic foot infections 2016–2021 (n = 637) Table 1 long description.

Variables	Values
**Age**, years (mean ± SD)	59 ± 13
**Sex**, male, n (%)	448 (70)
**Ethnicity**, n (%)	
Spanish, Hispanic or Latino	326 (53)
Not Spanish, Hispanic or Latino	293 (47)
**Race**, n (%)	
Black or African American	183 (30)
White	76 (12)
Asian, American Indian or Alaska Native	9 (1.5)
Other	341 (56)
**Diabetes type**, n (%)	
Type 1 diabetes	29 (5)
Type 2 diabetes	603 (95)
**Diabetes duration,** years (median, IQR)	11 (6–20)
**HbA1c**, % (mean ± SD)	9.8 ± 2.5
**Culture type**	
Blood culture, n (%)	539 (85)
Wound culture, n (%)	450 (71)
**Amputation during admission**, n (%)	283 (44)
**Length of stay**, days (median, IQR)	7 (4–11)

### Microbiologic testing and sampling practices

**Figure 2.**
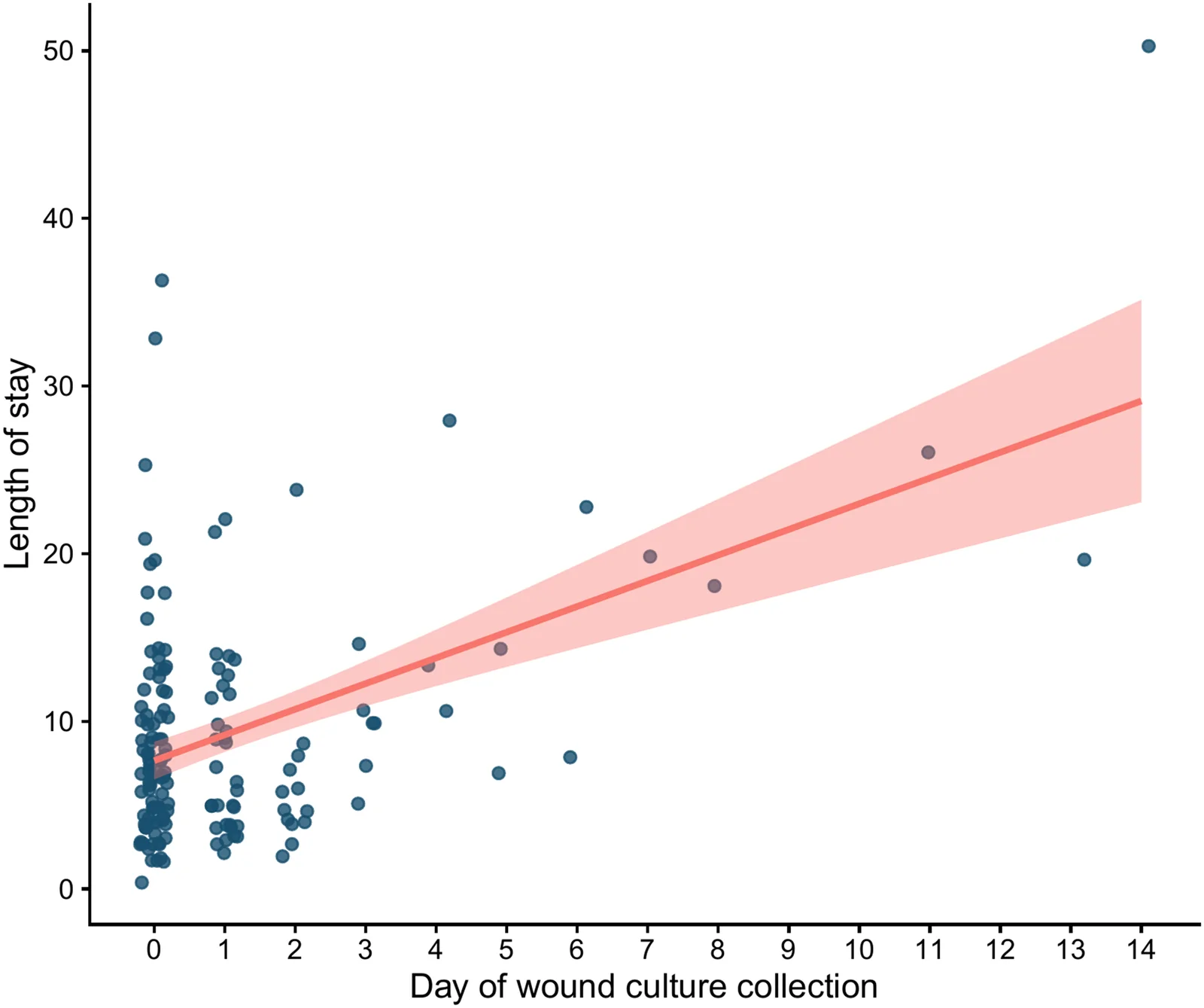
Association between wound culture sampling time and length of hospital stay. Scatter plot showing the relationship between timing of first wound culture collection (x axis; day 0 = hospital admission) and length of hospital stay (y axis). Each point represents an individual patient. The red line indicates the fitted linear regression, and the shaded area represents the 95% confidence interval. Analysis was restricted to patients admitted from August 2018 onward, when wound culture collection dates became available in the EHR (n = 202).

Blood cultures were obtained in 539 patients (85%), whereas wound cultures (including swabs, abscess aspirates, fluid or tissue specimens) were obtained in 450 patients (71%) (Figure 1, Table 1). Among patients with available wound culture timing data (n = 202), the median time to first sampling was 1 day (IQR 0–2). However, 37 patients (18%) experienced delayed sampling beyond 48 hours (Figure 2).

Delayed wound culture acquisition was associated with longer hospitalization. Time to first culture positively correlated with LOS (Pearson *r* = .47, 95% CI .35–.57, *P* < .001). In linear regression, each additional day from admission to culture collection was associated with a 1.18-day increase in LOS (β = 1.18, *P* < .001) (Figure 2).

### Wound culture results

A total of 2,397 wound cultures were obtained during hospitalization with a mean of 5.3 cultures per patient (Figure 1). Of these, 333 (13.9%) were tissue biopsies. Growth of bacteria or fungi was detected in 1,184 samples (49.4%). After exclusion of 150 samples (6.3%) considered colonizers, the final data set included 883 positive wound culture results. Polymicrobial infections were identified in 246 (27%) of positive cultures. Organisms identified in ≤ 5 patients were excluded from the primary analysis and are reported in Supplementary Table 1.

**Figure 3.**
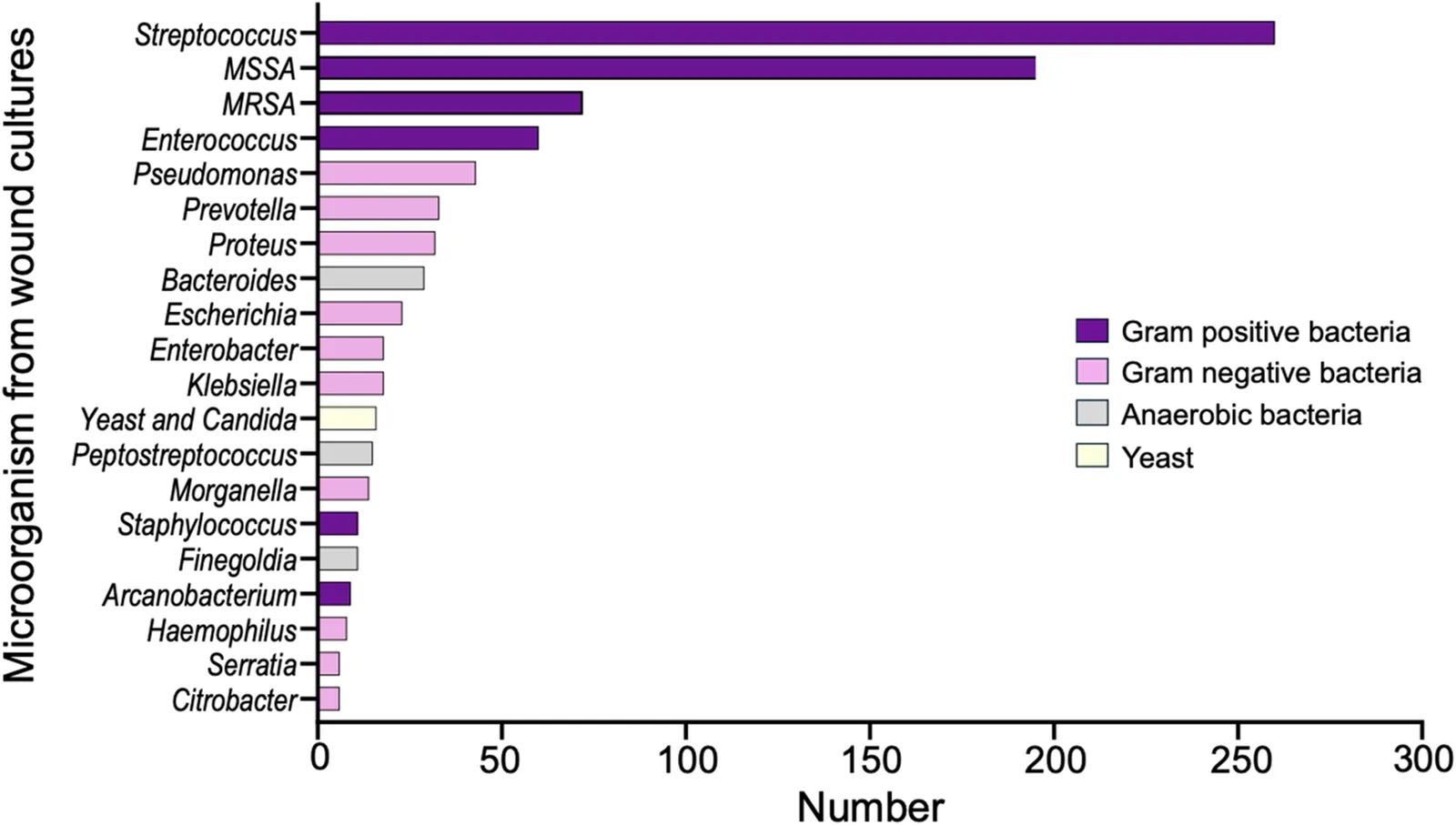
Pathogens isolated from diabetic foot infection (DFI) wound cultures. Bacteria and yeast recovered from wound specimens (swabs, abscess aspirates, fluid, or tissue). If the same organism was isolated from multiple samples in a single patient, it was counted once per patient. MSSA, Methicillin sensitive Staphylococcus aureus; MRSA, Methicillin resistant Staphylococcus aureus.

Among isolates, 611 (69%) were aerobic Gram-positive bacteria, 203 (23%) were Gram negative bacteria and 53 (6%) were facultative anaerobes. *S. aureus* was the most frequently isolated organism, identified in 274 (31%) cultures; of these, 200 (73%) were methicillin-susceptible *S. aureus* (MSSA), and 74 (27%) were methicillin resistant *S. aureus* (MRSA). Overall, MRSA accounted for 8% of all positive wound cultures, whereas *Pseudomonas aeruginosa* was isolated in 5% (Figure 3).

### Blood culture results

**Figure 4.**
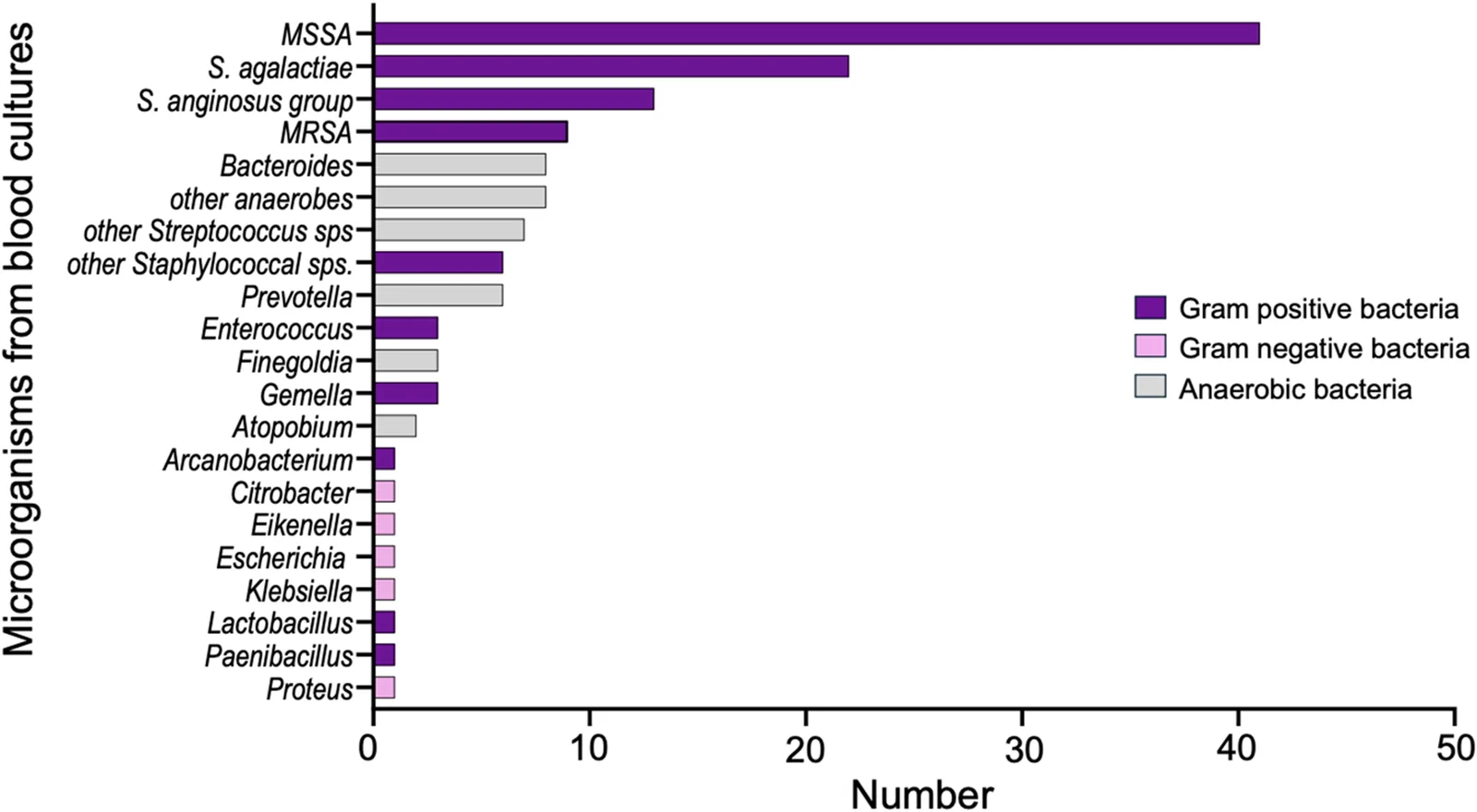
Blood culture results of patients hospitalized with diabetic foot infection (DFI). Only the first positive blood culture per patient is included. MSSA, Methicillin sensitive Staphylococcus aureus; MRSA, Methicillin resistant Staphylococcus aureus.

Among 539 patients with blood cultures, 139 (26%) had at least one positive blood culture (Figure 4). *S. aureus* was the most common bloodstream pathogen, identified in 50 patients (36% of bacteremia cases), including 41 (29.5%) MSSA, and 9 (6.4%) MRSA isolates. Other organisms included *S. epidermidis* (n = 4) and *S. hominis* (n = 2). Facultative anaerobes were identified in 28 patients (20%), most commonly *Bacteroides* and *Prevotella* species. No cases of *P. aeruginosa* bacteremia were detected.

Among patients with positive blood cultures, 30 (22%) had concordant organisms in wound cultures, 52 (37%) had discordant results, and 57 (41%) had bacteremia with sterile wound cultures.

### Antibiotic exposure and utilization patterns

The cohort had 19,039 inpatient pharmacy antibiotic administration orders. After excluding topical fluoroquinolone eye drops (n = 156, .8%), oral vancomycin (n = 97, .5%), and topical antibiotics (n = 375, 2.0%), a total of 18,411 systemic orders were included in the analysis.

**Figure 5.**
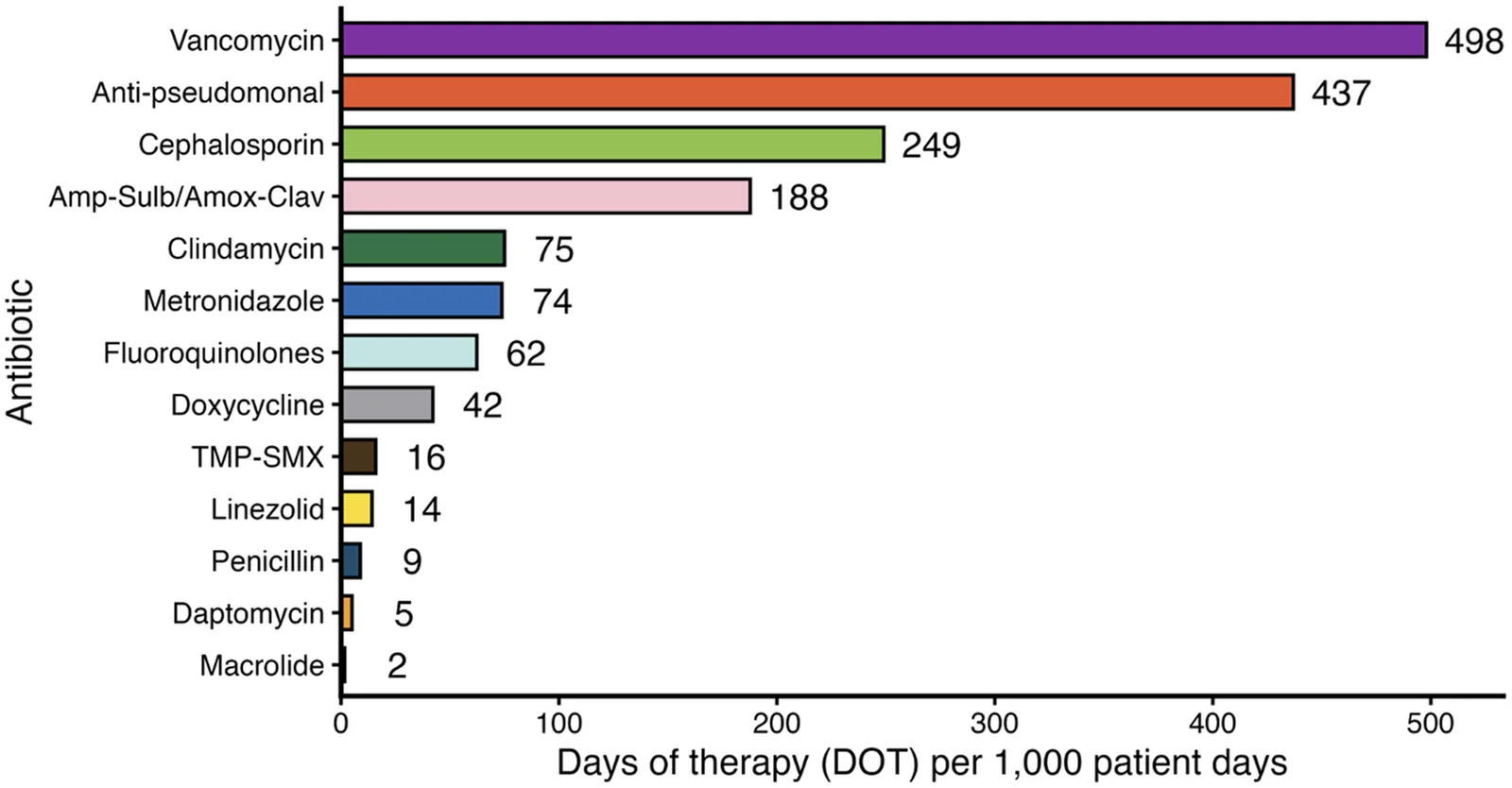
Distribution of antibiotic exposure by class in hospitalized patients with diabetic foot infection. Days of therapy (DOT) per 1,000 patient-days stratified by antibiotic class. DOT is defined as the number of days a patient receives an antibiotic, regardless of dose frequency, standardized by patient days. Cephalosporins shown represent agents without anti-pseudomonal activity. Anti-pseudomonal agents (shown separately) include cefepime, piperacillin-tazobactam, meropenem, aztreonam, and gentamicin. Abbreviations: Amp-Sulb, ampicillin-sulbactam; Amox-Clav, amoxicillin-clavulanic acid; TMP-SMX, trimethoprim/sulfamethoxazole.

Overall antibiotic exposure was 1,695 DOT per 1,000 patient-days and 961 LOT per 1,000 patient-days. DOT was highest for vancomycin, anti-pseudomonal agents, and non-antipseudomonal cephalosporins (Figure 5). By AWaRe classification, most antibiotic use fell within the Watch category (789 DOT per 1,000 patient-days), followed by Access (437) and Reserve agents (31). The median duration of inpatient antibiotic therapy was 7 days (IQR, 5–11).

Empiric therapy included broad spectrum coverage, with anti-MRSA agents administered to 581 patients (91%) and anti-pseudomonal agents to 512 patients (80%). Median duration of empiric anti-MRSA and anti-pseudomonal therapy was 4 days (IQR, 3–4) and 4 days (IQR, 3–7), respectively.

### Inpatient antibiotic de-escalation

**Figure 6.**
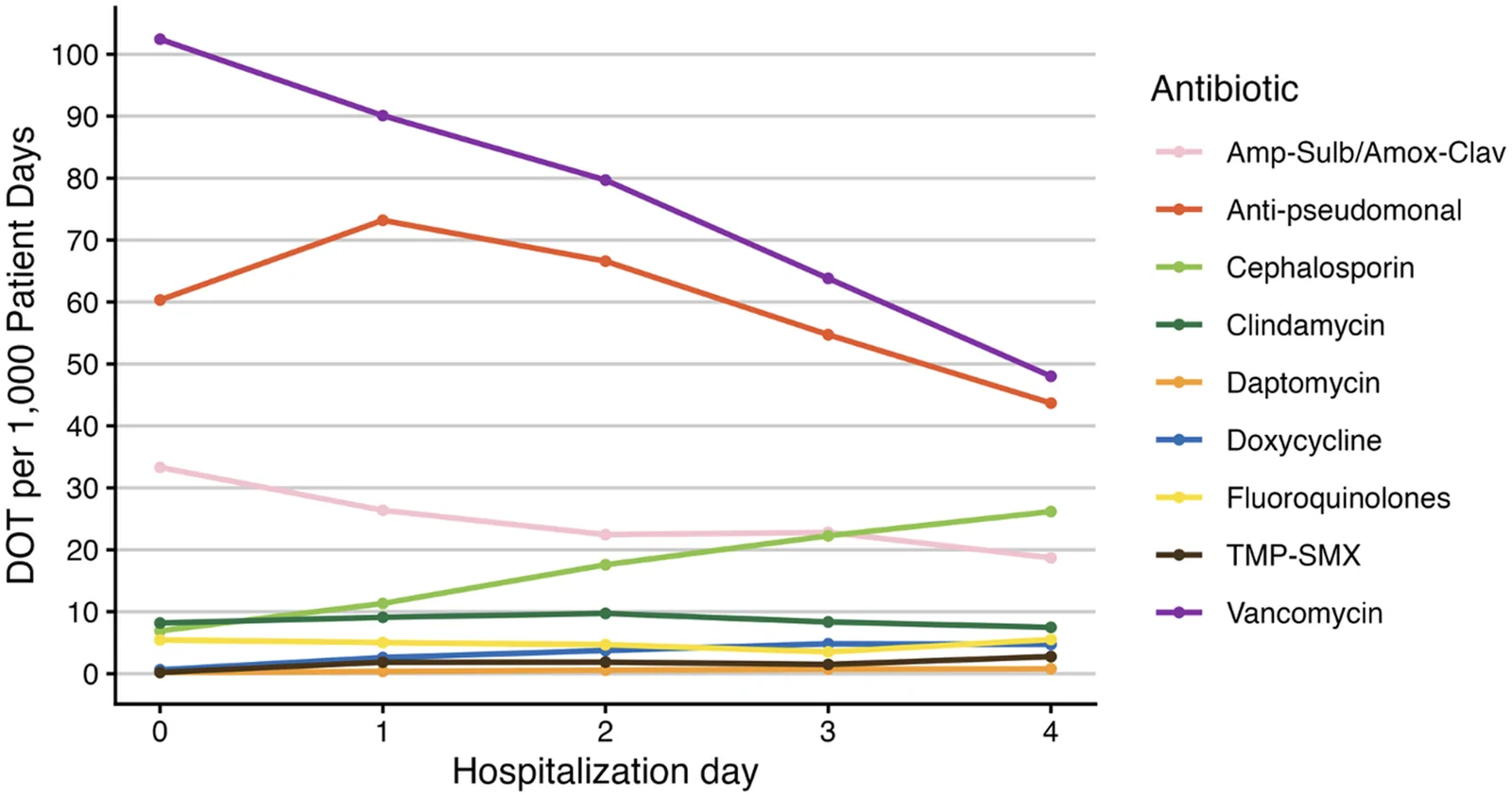
In-hospital trends in antibiotic use over time. Changes in antibiotic prescribing over the course of hospitalization. Each line represents modeled trends in days of therapy (DOT) per 1,000 patient-days, by antibiotic class. Only antibiotics showing a statistically significant change over time in linear regression models are shown. Cephalosporins shown represent agents without anti-pseudomonal activity. Anti-pseudomonal agents (shown separately) include cefepime, piperacillin-tazobactam, meropenem, aztreonam, and gentamicin. Abbreviations: Amp-Sulb, ampicillin-sulbactam; Amox-Clav, amoxicillin-clavulanic acid; TMP-SMX, trimethoprim/sulfamethoxazole.

Antibiotic regimens changed during hospitalization, consistent with de-escalation based on microbiologic data. (Figure 6 Supplement Table 2). Use of vancomycin (β = −1.3, 95% CI −1.8, −.9), anti-pseudomonal agents (β = −.7, 95% CI −.9, −.4), and cephalosporins (β = −.3, 95% CI −.4, −.2) significantly decreased over time during hospitalization. In contrast, use of fluoroquinolones, daptomycin, TMP-SMX, and clindamycin increased (*P* < .001), as did doxycycline (*P* = .04) (Supplementary Table 2).

### Temporal trends over five years

**Figure 7.**
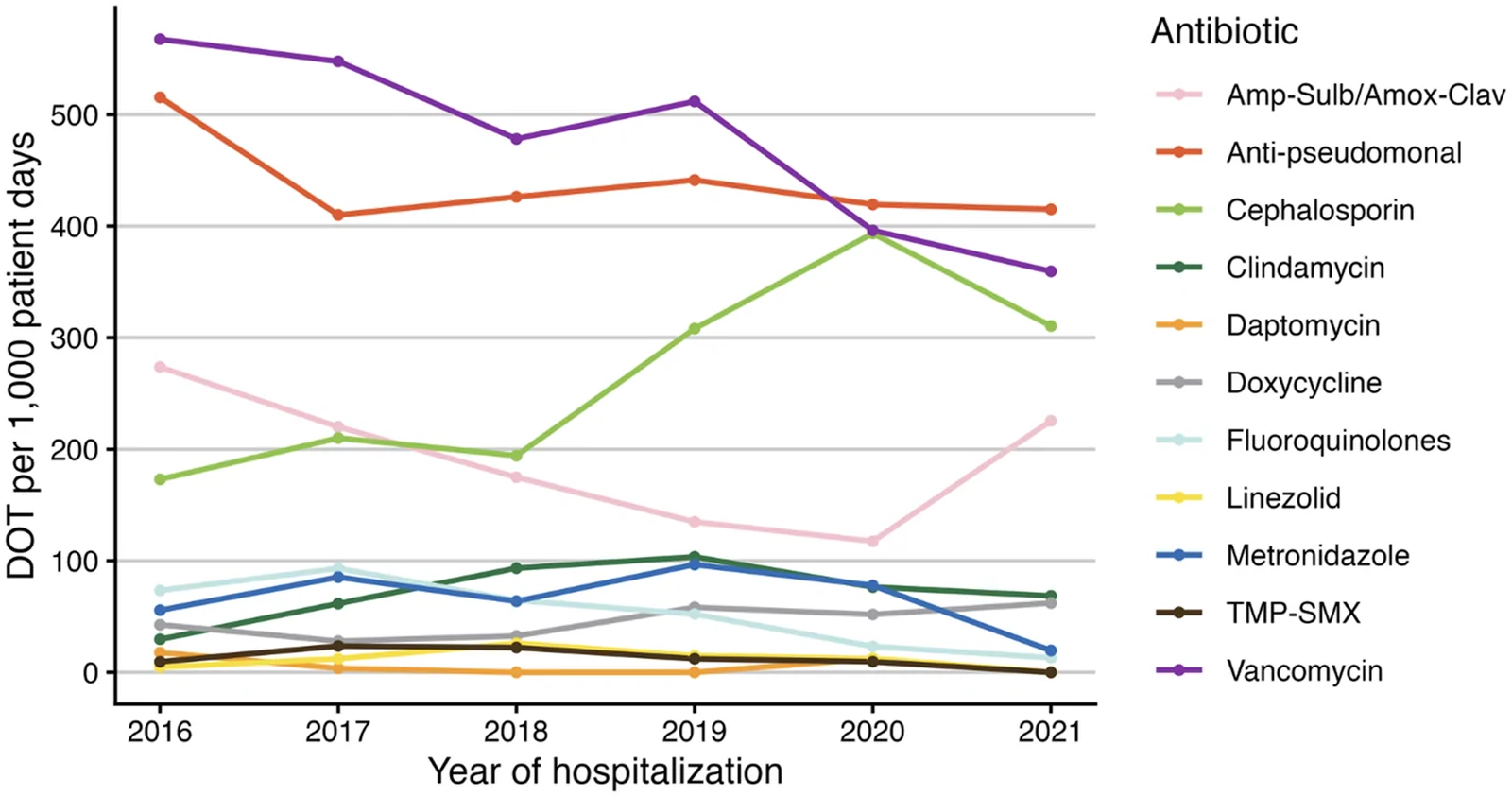
Temporal trends in antibiotic use over the five-year study period. Annual changes in antibiotic use by class from 2016 to 2021. Each line represents days of therapy (DOT) per 1,000 patient-days. Cephalosporins displayed in the figure represent those without anti-pseudomonal activity. Anti-pseudomonal agents (shown separately) include cefepime, piperacillin-tazobactam, meropenem, aztreonam, and gentamicin. Abbreviations: Amp-Sulb, ampicillin-sulbactam; Amox-Clav, amoxicillin-clavulanic acid; TMP-SMX, trimethoprim/sulfamethoxazole.

Across the five-year study period, there was a significant decrease in use of vancomycin (β = −41.7, 95% CI −63.4 to −20.1) and fluoroquinolones (β = −15.0, 95% CI −23.9 to −6.1), and a significant increase in use of non-antipseudomonal cephalosporins (β = +38.1, 95% CI 4.1 to 74.1) (Figure 7, Supplementary Table 3). No significant temporal changes were observed in other antibiotic classes (Supplementary Table 3). Trends stratified by AWaRe classification are presented in the Supplementary Table 4 and Supplementary Figure 1.

## Discussion

In this large retrospective cohort of hospitalized patients with DFIs, we found a consistent pattern of discordance between local microbiologic epidemiology and empiric antibiotic prescribing. Although *S. aureus*—predominantly MSSA—was the most common pathogen in both wound and blood cultures, and *P. aeruginosa* and MRSA were infrequently identified, empiric therapy frequently included broad-spectrum anti-MRSA and antipseudomonal coverage. These findings suggest substantial opportunities for antimicrobial stewardship optimization in inpatient DFI management.

A key finding of this study is the association between delayed wound culture acquisition and longer LOS.^13^ Although causality cannot be established, this relationship likely reflects a combination of clinical and system-level factors. Delayed sampling may postpone organism identification and subsequent de-escalation, potentially prolonging broad-spectrum antibiotic exposure and hospitalization. Alternatively, patients with more complex or severe infections may both require longer hospitalization and experience delays in diagnostic sampling. Regardless, our findings underscore the importance of early, high-quality specimen collection as a potential target for improving diagnostic efficiency and resource utilization.^14,15^

Our microbiologic findings are consistent with prior studies demonstrating that DFIs are most commonly caused by Gram-positive organisms, particularly *S. aureus*, with variable contributions from streptococci and Gram-negative organisms. Importantly, MRSA and *P. aeruginosa* were uncommon in both wound and blood cultures, reinforcing prior evidence that routine empiric coverage for these organisms may not be necessary in many North American settings.^14,17^ Despite this, empiric anti-MRSA and antipseudomonal therapy was administered to the vast majority of patients, typically for several days before de-escalation. Although we observed an encouraging decline in vancomycin use over time and evidence of inpatient de-escalation, overall empiric exposure to broad-spectrum agents remained high.

These prescribing patterns have important implications for antimicrobial stewardship. Excessive empiric use of broad-spectrum antibiotics, particularly agents classified in the WHO “Watch” category, may contribute to selection pressure and antimicrobial resistance.^18^ In our cohort, most of antibiotic exposure fell within this category, highlighting an area for targeted intervention. Potential strategies include earlier de-escalation protocols, enhanced use of rapid diagnostics such as MRSA nasal PCR testing, and stronger alignment of empiric therapy with local microbiologic data.^16^

We also observed a shift in antibiotic use over time, including decreased use of vancomycin and fluoroquinolones and increased use of non-antipseudomonal cephalosporins. These trends may reflect evolving stewardship practices and increased awareness of resistance risks. However, the lack of a corresponding reduction in antipseudomonal prescribing suggests persistent diagnostic uncertainty or entrenched prescribing habits in initial empiric regimens.

Our study has several strengths. It includes a large, diverse urban cohort with detailed microbiologic and prescribing data over a five-year period. The integration of culture data with granular antibiotic utilization metrics (DOT, LOT, and AWaRE classification) provides a comprehensive assessment of inpatient antimicrobial use patterns in DFIs. Additionally, evaluation of both within-hospital and longitudinal prescribing trends offers insight into dynamic prescribing behavior.

Several limitations should be acknowledged. We defined DFI based on antibiotic exposure, which may have misclassified some cases, overestimated true infection burden, or reflected treatment of infections other than DFI. As a single hospital system study, generalizability to other healthcare settings with different patient populations or prescribing practices may be limited. We did not directly assess appropriateness of individual antibiotic choices, link specific pathogens to prescribing decisions at the patient level, or evaluate antibiotic allergies, which may have influenced antibiotic selection. Details regarding the collection method and setting of tissue specimens were not consistently available, limiting our ability to distinguish operative specimens from those obtained by other techniques and potentially resulting in misclassification of some organisms as pathogenic. Finally, restricting analysis to index hospitalizations may not capture recurrence patterns or longitudinal outcomes in patients with recurrent DFIs.

Beyond antimicrobial stewardship, our findings have important implications for hospital practice. The discrepancy between local microbiologic epidemiology and empiric antibiotic prescribing highlights an opportunity to develop institution-specific treatment pathways that incorporate local susceptibility data while accounting for individual patient risk factors. Standardized approaches to early wound culture acquisition, combined with antimicrobial stewardship review and timely reassessment of empiric therapy, may reduce unnecessary exposure to broad-spectrum antibiotics without compromising appropriate initial management. Future studies should evaluate whether implementation of these stewardship and diagnostic strategies improves clinical outcomes and antibiotic utilization while also examining whether patients undergoing amputation have distinct microbiologic and antibiotic use patterns that could inform more tailored management.

## Conclusions

In conclusion, we demonstrate a persistent mismatch between microbiologic epidemiology and empiric antibiotic prescribing in hospitalized DFIs. Despite low rates of MRSA and *P. aeruginosa*, broad-spectrum empiric therapy remains common, although inpatient de-escalation is frequent and overall vancomycin use has declined over time. These findings highlight important opportunities for antimicrobial stewardship interventions focused on earlier culture acquisition, improved diagnostic precision, and more targeted empiric therapy aligned with local epidemiology.

## Supporting information

10.1017/ash.2026.10824.sm001 Milosavljevic et al. supplementary material 1 Milosavljevic et al. supplementary material

10.1017/ash.2026.10824.sm002 Milosavljevic et al. supplementary material 2 Milosavljevic et al. supplementary material

## Supplementary material

The supplementary material for this article can be found at https://doi.org/10.1017/ash.2026.10824.

## Table 1. Long description

A table with 12 rows and 2 columns detailing demographic and clinical characteristics of patients hospitalized with diabetic foot infections. The columns are labeled ‘Variables’ and ‘Values’. The table includes the following data: Row 1: Age, years (mean ± SD), 59 ± 13. Row 2: Sex, male, n (%), 448 (70). Row 3: Ethnicity, n (%), Spanish, Hispanic or Latino, 326 (53). Row 4: Not Spanish, Hispanic or Latino, 293 (47). Row 5: Race, n (%), Black or African American, 183 (30). Row 6: White, 76 (12). Row 7: Asian, American Indian or Alaska Native, 9 (1.5). Row 8: Other, 341 (56). Row 9: Diabetes type, n (%), Type 1 diabetes, 29 (5). Row 10: Type 2 diabetes, 603 (95). Row 11: Diabetes duration, years (median, IQR), 11 (6–20). Row 12: HbA1c, % (mean ± SD), 9.8 ± 2.5. Row 13: Culture type, Blood culture, n (%), 539 (85). Row 14: Wound culture, n (%), 450 (71). Row 15: Amputation during admission, n (%), 283 (44). Row 16: Length of stay, days (median, IQR), 7 (4–11).

Navigate back to Table 1..
