# Supplementary material for: Five-year trends in antibiotic use and microbiology of hospitalized diabetic foot infections in the Bronx
Source: Antimicrob Steward Healthc Epidemiol. 2026 Sep 9;6(1):e262. doi: 10.1017/ash.2026.10824 (PMC13760296; doi:10.1017/ash.2026.10824)
Supplement: Supplementary file 1 [file S2732494X26108249sup002.docx]

| **Supplementary Table 1. Rarely identified species** | |
| --- | --- |
| **Species** | **No. of patients isolated** |
| *Acinetobacter baumannii/calcoaceticus complex* | 1 |
| *Acinetobacter radioresistens* | 1 |
| *Actinomyces neuii* | 1 |
| *Actinomyces neuii ssp neuii* | 1 |
| *Actinomyces radingae* | 1 |
| *Actinomyces turicensis* | 1 |
| *Bacteroides stercoris* | 1 |
| *Bacteroides uniformis* | 1 |
| *Bifidobacterium bifidum* | 1 |
| *Candida parapsilosis* | 1 |
| *Citrobacter freundii* | 1 |
| *Clostridium perfringens* | 1 |
| *Eikenella corrodens* | 1 |
| *Enterobacter* | 1 |
| *Enterococcus faecium* | 1 |
| *Enterococcus raffinosus* | 1 |
| *Gemella morbillorum* | 1 |
| *Haemophilus haemolyticus* | 1 |
| *Klebsiella pneumoniae ssp ozaenae* | 1 |
| *Kocuria* | 1 |
| *Lactococcus* | 1 |
| *Myroides odoratimimus* | 1 |
| *Neisseria mucosa* | 1 |
| *Pantoea agglomerans* | 1 |
| *Pasteurella canis* | 1 |
| *Pasteurella multocida* | 1 |
| *Peptoniphilus* | 1 |
| *Prevotella buccae* | 1 |
| *Proteus hauseri* | 1 |
| *Proteus penneri* | 1 |
| *Providencia stuartii* | 1 |
| *Serratia liquefaciens* | 1 |
| *Staphylococcus capitis* | 1 |
| *Staphylococcus chromogenes* | 1 |
| *Staphylococcus cohnii* | 1 |
| *Staphylococcus pettenkoferi* | 1 |
| *Staphylococcus schleiferi* | 1 |
| *Streptococcus equinus* | 1 |
| *Streptococcus salivarius* | 1 |
| *Trichosporon asahii* | 1 |
| *Vagococcus fluvialis* | 1 |
| *Veillonella* | 1 |
| *Alcaligenes faecalis* | 2 |
| *Arcanobacterium bernardiae* | 2 |
| *Bacteroides capillosus* | 2 |
| *Bacteroides ovatus* | 2 |
| *Bacteroides pyogenes* | 2 |
| *Beta Streptococcus group A* | 2 |
| *Bifidobacterium dentium* | 2 |
| *Candida glabrata* | 2 |
| *Candida tropicalis* | 2 |
| *Dermabacter hominis* | 2 |
| *Fusarium* | 2 |
| *Helcococcus kunzii* | 2 |
| *Stenotrophomonas maltophilia* | 2 |
| *Streptococcus intermedius* | 2 |
| *Vancomycin-Resistant Enterococcus faecalis (VRE)* | 2 |
| *Actinomyces* | 3 |
| *Atopobium parvulum* | 3 |
| *Bacteroides caccae* | 3 |
| *Bacteroides distasonis* | 3 |
| *Bacteroides vulgatus group* | 3 |
| *Enterobacter aerogenes* | 3 |
| *Prevotella denticola* | 3 |
| *Proteus vulgaris/penneri* | 3 |
| *Streptococcus dysgalactiae* | 3 |
| *Streptococcus oralis* | 3 |
| *Actinomyces odontolyticus* | 4 |
| *Bacteroides vulgatus* | 4 |
| *Prevotella* | 4 |
| *Prevotella intermedia* | 4 |
| *Staphylococcus caprae* | 4 |
| *Staphylococcus simulans* | 4 |
| *Bacteroides thetaiotaomicron* | 5 |
| *Bacteroides thetaiotaomicron* group | 5 |
| *Beta Streptococcus* group C | 5 |
| *Peptostreptococcus anaerobius* | 5 |
| *Proteus vulgaris* | 5 |
| Organisms identified in 5 or less patients were classified as rare organisms. | |

| **Supplementary Table 2.** Daily change in DOT per 1,000 patient-days per each admission day | | |
| --- | --- | --- |
| **Antibiotic Class** | **Mean Change in DOT per 1,000 patient-days** | **p-value** |
| **Ampicillin-sulbactam/Amoxicillin-clavulanic acid** | -0.02 (-0.17, 0.13) | 0.77 |
| **Anti-pseudomonal agents** | -0.69 (-0.92, -0.45) | <0.001 |
| **Cephalosporin without anti-pseudomonal coverage** | -0.30 (-0.44, -0.17) | <0.001 |
| **Clindamycin** | 0.15 (0.07, 0.24) | <0.001 |
| **Daptomycin** | 0.14 (0.10, 0.18) | <0.001 |
| **Doxycycline** | 0.08 (0.01, 0.16) | 0.04 |
| **Fluoroquinolones** | 0.12 (0.07, 0.19) | <0.001 |
| **Linezolid** | 0.01 (-0.04, 0.07) | 0.70 |
| **Macrolide** | -0.01 (-0.06, 0.03) | 0.51 |
| **Metronidazole** | 0.05 (-0.01, 0.11) | 0.13 |
| **Penicillin** | 0.01 (-0.04, 0.06) | 0.69 |
| **Trimethoprim/sulfamethoxazole** | 0.11 (0.05, 0.17) | <0.001 |
| **Vancomycin** | -1.35 (-1.80, -0.90) | <0.001 |
| Abbreviations: DOT - days of therapy. Daily mean and 95% CI change in DOT per 1,000 patient-days for each antibiotic class were estimated by simple linear regression with hospitalization day as independent and DOT as dependent variable. Anti-pseudomonal agents include cefepime, piperacillin-tazobactam, meropenem, aztreonam, gentamicin. | | |

| **Supplementary Table 3.** Annual Change in DOT per 1,000 patient-days | | |
| --- | --- | --- |
| **Antibiotic Class** | **Mean Change in DOT per 1,000 patient-days** | **p-value** |
| **Ampicillin-sulbactam/Amoxicillin-clavulanic acid** | -16.82 (-54.26, 20.62) | 0.28 |
| **Anti-pseudomonal agents** | -13.12 (-36.07, 9.86) | 0.19 |
| **Cephalosporin without anti-pseudomonal coverage** | 38.61 (4.08, 73.14) | 0.04 |
| **Clindamycin** | 7.14 (-9.45, 23.72) | 0.29 |
| **Daptomycin** | -0.38 (-43.79, 43.02) | 0.93 |
| **Doxycycline** | 5.56 (-1.23, 12.36) | 0.08 |
| **Fluoroquinolones** | -14.97 (-23.90, -6.05) | 0.01 |
| **Linezolid** | 1.72 (-6.52, 10.07) | 0.54 |
| **Macrolide** | 0.11 (-1.44, 1.65) | 0.79 |
| **Metronidazole** | -4.85 (-23.92, 14.21) | 0.52 |
| **Penicillin** | -0.47 (-3.69, 2.74) | 0.70 |
| **Trimethoprim/sulfamethoxazole** | -1.13 (-8.96, 6.70) | 0.68 |
| **Vancomycin** | -41.75 (-63.37, -20.13) | 0.006 |
| Abbreviations: DOT - days of therapy. Annual mean and 95% CI change in DOT per 1,000 patient-days for each antibiotic class were estimated by simple linear regression with hospitalization year as independent and DOT as dependent variable. Anti-pseudomonal agents include cefepime, piperacillin-tazobactam, meropenem, aztreonam, gentamicin. | | |

| **Supplementary Table 4.** Annual Change in DOT per 1,000 patient-days | | |
| --- | --- | --- |
| **AWaRE Group** | **Mean Change in DOT per 1,000 patient-days** | **p-value** |
| **Access** | 5.01 (-7.84, 18.01) | 0.34 |
| **Watch** | -41.67 (-83.21, -0.13) | 0.049 |
| **Reserve** | -3.67 (-10.04, 2.71) | 0.19 |
| Abbreviations: DOT - days of therapy. Annual mean and 95% CI change in DOT per 1,000 patient-days for each AWaRe class were estimated by simple linear regression with hospitalization year as independent and DOT as dependent variable. | | |

**Supplementary Figure 1. Trends in antibiotic use over time 2016-2021 in patients hospitalized with DFI by AWaRe antibiotic class.** Each line represents days of therapy (DOT) per 1,000 patient-days.
